# Localizing, describing, interpreting: effects of different audio text structures on attributing meaning to digital pictures

**DOI:** 10.1007/s11251-022-09593-6

**Published:** 2022-08-11

**Authors:** Manuela Glaser, Manuel Knoos, Stephan Schwan

**Affiliations:** 1 Leibniz-Institut für Wissensmedien, Schleichstr. 6, 72076 Tuebingen, Germany; 2 Im Bruckenschlegel 7, 70186 Stuttgart, Germany

**Keywords:** Audio text coherence, Multimedia, Dual coding, Picture comprehension, Visual attention

## Abstract

Based on previous research on multimedia learning and text comprehension, an eye-tracking study was conducted to examine the influence of audio text coherence on visual attention and memory in a multimedia learning situation with a focus on picture comprehension. Audio text coherence was manipulated by the type of LDI structure, that is, whether localization, description, and interpretation followed in immediate succession for each pictorial detail or whether localizations and description of details were separated from their interpretation. Results show that with a LDI integrated structure compared to a LDI separated structure the referred-to picture elements were fixated longer during interpretation parts, and linkages between descriptions and interpretations were better recalled and recognized. The effects on recall and recognition of linkages were fully mediated by fixation times. This pattern of results can be explained by an interplay between audio text coherence and dual coding processes. It points out the importance of local coherence and the provision of localization information in audio explanations as well as visual attention to allow for dual coding processes that can be used to better attribute meaning to picture details. Practical implications for the design of educational videos, audio texts on websites, and audio guides are discussed.

## Introduction

Complex digital pictures such as maps, satellite pictures, x-ray visualizations, or paintings often cannot be adequately understood without additional information that directs the viewer’s gaze to important elements of the picture (Cho, 2016; Lupyan & Ward, 2013) and to help them interpret what they see. Therefore, in multimedia learning scenarios such as online courses, educational software, internet search, television, or museum visits, complex pictures are often presented with accompanying audio texts explaining them (Leahy et al., 2003; Kalyuga, 2012). In online courses, educational software, and internet search, such audio explanations are provided and regularly used via audio files or audio tracks within videos or video podcasts. In particular, educational videos and video podcasts are used and examined more and more in schools, in online courses, as well as on internet platforms such as YouTube or Vimeo (Kay, 2012; Shoufan, 2019). The German X-ray Society (Deutsche Röntgengesellschaft), for example, provides an online course on its YouTube Channel about the correct interpretation of x-ray tomography images for students preparing for their exams.

In museums, audio explanations are typically provided, used, and examined in the form of audio guides or digital guides (Sung et al., 2010). Furthermore, beyond on-site museum visits, more and more museums provide pictures, high-resolution images, or 3D models of their exhibits or even whole collections together with accompanying explanations in the form of audio files or videos on their websites. One such example is the Metropolitan Museum of Art of New York City (https://www.metmuseum.org), which provides on its website, among other things, videos by which recipients can join curators on guided tours through the galleries as well as pictures of paintings with accompanying audio explanations in which experts describe and explain the paintings. Furthermore, the Metropolitan Museum of Art of New York City (https://www.youtube.com/user/metmuseum) has – like many other museums such as the National Gallery (https://www.youtube.com/user/nationalgalleryuk) and the Tate Gallery of Modern Art in London (https://www.youtube.com/user/tate) or the Louvre in Paris (https://www.youtube.com/user/louvre) – its own YouTube channel on which it also provides videos that take the viewers on a tour through the exhibitions, thereby explaining paintings or other exhibits. Important to mention is also the virtual archive Europeana (https://www.europeana.eu), which aims to make the scientific and cultural heritage of Europe - from prehistory and early history to the present - available to a broad public in the form of image, text, sound, and video files as well as 3D models.

These examples show that in a number of contexts of knowledge transfer pictures have priority over texts and, accordingly, that the effects of accompanying audio explanations on picture processing are of high practical relevance. If such audio explanations are appropriately formulated, they may guide the viewers’ attention through the picture, help them to notice important elements, establish semantic and formal relations, and make meaning of what is presented in the picture (Schwan et al., 2018; Webb & Mann, 2014). But how can these audio explanations be designed appropriately?

Multimedia research provides basic empirical research on how to combine written or spoken texts with pictures (Mayer, 2014). However, it deals mostly with learning scenarios having the main focus on texts in which pictures are merely used as accompanying illustrations and not vice versa. But recently, several studies have also examined multimedia learning with a focus on pictures (Glaser & Schwan, 2015, 2020; Schnotz et al., 2021; Xie et al., 2018). In addition, multimedia research has focused on how to design combinations of text and picture information according to principles such as the multimedia principle (Hu et al., 2021), the modality principle (Ginns, 2005), the spatial and the temporal contiguity principle (Ginns, 2006), or the cueing principle (Richter et al., 2016), but research on how to formulate audio explanations in order to foster knowledge acquisition in multimedia learning situations is scarce (Glaser et al., 2020a, b).

An important aspect of audio explanation and educational video design is text coherence (Kulgemeyer, 2020). In the present context, text coherence means that an audio explanation is designed in a way that signals the global structure of the audio text as well as local linguistic connections between sentences and corresponding arguments and thereby assists an explainee in constructing a mental representation of the explained content. Research has shown that text coherence facilitates text comprehension especially for learners with low prior knowledge (McNamara et al., 1996) – this interaction with prior knowledge being present for processing informative but not for persuasive texts (Kamalski et al., 2008). However, until now text coherence has been empirically investigated mainly with written texts alone. In contrast, only a few studies have dealt with the effect of coherence of audio texts (Carroll & Korukina, 1999), picture stories (Cohn, 2014; Hagmann & Cohn, 2016; Hutson et al., 2018), or combinations of spoken text and pictures (Tibus et al., 2013) on recipients’ comprehension. The present study therefore examines the influence of audio text coherence on attention and memory in the multimedia context of picture comprehension with accompanying audio explanations.

## Localizing, Describing, Interpreting (LDI) as Basic Structures of Audio Explanations of Pictures

As part of the cognitive theory of multimedia learning (CTML) by Mayer (2009, 2014), the multimedia principle assumes that the linking of text information with corresponding picture information leads to better comprehension than the sole presentation of the text information (Butcher, 2014; Mayer, 2009). This assumption is based on Paivio’s (1971) dual coding theory which posits that visual and verbal information are processed along different channels in the human cognitive system and that the ability to code a multimodal stimulus in two complementary ways increases the probability of remembering it compared to when the stimulus is only coded one way. The multimedia principle assigns to pictures an illustrative and comprehension-supporting function for text understanding in accordance with basic approaches to text-picture integration (Anglin et al., 2004). The complementary notion that texts have a supporting function for the processing and understanding of pictures and that the linking of picture information to corresponding text information leads to better comprehension than the sole presentation of the picture information has also been validated empirically (Glaser & Schwan, 2015, 2020; Huff & Schwan, 2008, 2012; Mayer & Anderson, 1992). Regarding multimedia effects on picture comprehension, Glaser and Schwan (2015) concluded that guiding visual attention by means of accompanying verbal references constitutes an important third mechanism, over and above dual coding and the reduction of mental load by presenting information in two modalities. Accordingly, empirical evidence demonstrates that adding verbal explanations to pictures increases fixation times on named pictorial elements, enhances memory of these elements, and leads to an inter-individual homogenization of gaze behaviour (Glaser & Schwan, 2015, 2020; Glaser et al., 2020a; Schnotz et al., 2021; Xie et al., 2018). This is in line with the eye-mind hypothesis, which states that eye-fixated content is simultaneously processed in working memory (Just & Carpenter, 1980) and which is at least in general supported by a lot of research (Holmqvist et al., 2011).

But while the supporting function of accompanying verbal explanations on picture comprehension has been shown in general, less is known how to design such texts appropriately. In other words: How should the audio track of a YouTube video explaining pictorial material be made in order to support comprehension? Which linguistic features of the accompanying verbal explanations facilitate the learning of visual depictions in multimedia online material?

Besides the mere naming of picture elements, audio explanations typically provide information on the spatial position of the picture elements, a description of their visual appearance, as well as an interpretation of them which is crucial for the viewers to make meaning of the picture. In the following, we will term this sequence of *l*ocalizing, *d*escribing, and *i*nterpreting as the LDI structure of a text explaining a picture. Localization information in an audio text is given by formulations such as “in the left corner of the painting” (localization in relation to the picture frame) or “above [a particular picture element]” (localization in relation to another previously mentioned picture element). Localization information is always related to certain picture elements or a group of picture elements. They inform viewers about the position of certain picture elements or certain pictorial areas. These picture elements can then be addressed by their description and/or their interpretation. Descriptions are formulations of the visual appearance or general semantic category of certain picture elements or areas such as “the dark blue area” (representation on a perceptual level) or “the lake” (representation on a schema-based low prior knowledge level). Descriptions can vary in their level of detail, for example “the lake”, “the dark blue lake”, or “the dark blue lake with its billowing waves”. Interpretations, on the other hand, are formulations of the meaning of particular picture elements such as “Loch Ness” (representation on a concrete level) or “justice” (representation on an abstract symbolic level). Providing localization information may help the viewers to direct their visual attention to the respective picture elements and foster dual coding, describing certain picture contents also may foster dual coding processes, and interpreting picture elements should make it easier for the viewers to integrate the cognitive representations of the respective picture elements to prior knowledge structures.

For example, a digital video on radiology may show an x-ray image of a chest, accompanied by a verbal explanation which guides the learner to a specific part of the image, describes a visual anomaly of the tissue at that position, interprets it as a certain type of tumour, and then proceeds to the next part of the image. Alternatively, the audio explanation of a historic painting available on the website of an art museum may first go systematically through the painting, localizing and describing all relevant pictorial elements before providing an overall interpretation of the painting’s content. The latter approach follows the stages of iconographic picture analysis formulated by Panofsky (1975), stating that a pictorial artwork should first be looked at closely and described exhaustively before the picture’s content is interpreted in a second, separate phase (Bauer & Schwan, 2018). It also follows notions of phenomenology to try first to observe a given phenomenon from an unbiased view before thinking about different interpretations and perspectives on this phenomenon in a second step (Eddles-Hirsch, 2015). Similarly, current teaching strategies in school subjects like history or literature emphasize the importance of asking students to first describe what they see without making any interpretation about what the picture is trying to say and then, in a second step, discuss the interpretation and message of the picture (Tishman, 2017). Accordingly, in an explanation of a picture, all relevant picture elements should first be localized and described - one after the other - and only then should the picture interpretation follow as a whole, whereby it is assumed that the viewer has already formed an appropriate mental representation of the picture in the first section of the explanation which is then linked to the interpretative statements of the second section. In this case, the description of the painting and the interpretative statements are separated from one another in time. In contrast, many audio guide explanations proceed by localizing, describing, and interpreting each individual picture element in immediate succession before moving on to the next picture element (Popp, 2013), similar to the radiology example described above.

This raises the until now not empirically examined question of which type of LDI structure is more suitable for picture comprehension: the integrated type of LDI structure, whereby each picture element is localized, described, and interpreted in direct succession before proceeding to the next element or the separated type of LDI structure, whereby all relevant picture elements are localized and described first before proceeding to their interpretation. Bringing together models of text comprehension and models of multimedia learning, it is to be expected that the cognitive processing differs systematically between these two LDI structure design variants of verbal explanations.

According to the construction-integration model of text comprehension by Kintsch (1988), texts are comprehended by activating word meanings, forming propositions, and producing inferences and elaborations, thereby creating a network of interrelated items which is then integrated into a coherent structure. In order to arrange a text’s propositions into a coherent mental model, bridging inferences and knowledge-based inferences are built. The more arguments overlap and the more related propositions are directly linked in the text - that is, the more coherent a text is - the less inferences are needed to establish a coherent mental model of its content. With regard to text coherence, local and global coherence has to be distinguished (van Dijk & Kintsch 1983). Local coherence is established when information currently being processed is linked to the immediately preceding context that is still present in the working memory. Global coherence, on the other hand, includes relations between currently processed information and information that was presented earlier in the text and is no longer in working memory (McKoon & Ratcliff, 1992). In this case, readers and listeners have to search their episodic text memory for possible related antecedents of the currently processed information and reset them into the working memory in order to relate both to each other and thereby understand the text. Therefore, texts that present related information in close proximity to one another in time (local coherence) should be easier to understand than texts that present related information at different times (global coherence). With regard to LDI structure this would mean that audio explanations with a LDI integrated structure rely on local coherence and should therefore be more easily comprehended than audio explanations with a LDI separated structure that require the more effortful establishing of global coherence and therefore impede fluent processing and comprehension.

In the case of a LDI integrated structure, the visual attention of the viewer during the interpretation phase is still directed to the corresponding picture element due to the initial verbal cueing in the localization and description passage. Hence, there should be a higher chance to link the given interpretation to the pictorial depiction of the element and therefore develop a dually coded integrated representation of the element’s visual appearance together with its description and interpretation. In the case of the temporal separation of description and interpretation (LDI separated), on the other hand, linking the visual appearance of the picture element to the accompanying verbal information should only be guaranteed for the descriptive part but not necessarily for the interpretative part. At the level of visual attention, this should be manifest in shorter fixations of the respective picture element during the interpretation part of the verbal information. At the level of memory and understanding, this should go along with a decreased memory for the interpretation and for the linkage between interpretation and visual appearance of the corresponding picture elements.

## The Present Study

In many multimedia learning scenarios such as online courses, educational software, internet search, television, or museum visits, complex pictures are often presented with accompanying audio texts explaining them. Since the role and influence of audio texts on picture processing and understanding in such situations has until now not been sufficiently examined, the present eye tracking study compares two different presentation formats with practical relevance for audio explanations accompanying pictures. Audio explanations either provide the localization and description of details of picture elements as well as their corresponding interpretations together for each picture element, which means that the descriptions of the details of a picture element are immediately followed by their interpretations (LDI integrated condition), or they provide the localization and description of the details of the picture elements as well as their corresponding interpretations separately, meaning that all picture elements are first localized and described with regard to their details and then the interpretation of all picture elements together follows (LDI separated condition). Based on multimedia theory and text comprehension research, we investigated how these two presentation formats affect visual attention, memory for the described details of the picture elements, memory for their interpretations, and memory for the linkages between the described details of the picture elements and their interpretation.

In the LDI integrated condition in which the details and interpretations are combined during the interpretation phase, the viewers should identify faster and fixate longer the referred-to picture elements and therefore be better able to link interpretations not only to the verbal description of the respective picture detail but additionally to the corresponding visual picture details itself. As a result, interpretations should be better stored in memory than in the LDI separated condition with separated detail description and interpretation inhibiting the possibility to connect the interpretation additionally to the visual picture detail. In this case, when presenting the interpretations, more visual search should be needed to identify and fixate the referred-to picture elements. Thus, during processing this interpretation, the possibility to connect interpretations with dually coded picture details and therefore memory for interpretations and the memory for linkages between details and their interpretations should be reduced. We preregistered the following hypotheses:

H1: Longer fixation times on the respective picture elements during the interpretation phases in the LDI integrated condition than in the LDI separated condition.

H2: Better memory of the interpretations in the LDI integrated condition than in the LDI separated condition.

H3: Better memory of linkages between the described details of the picture elements and their corresponding interpretations in the LDI integrated condition than in the LDI separated condition.

## Method

### Participants

Participants were recruited from our institute’s mailing list and received 12.50 Euros for their participation. They had to be native German speakers or have an equivalent level of fluency. Participants studying art, art history, or history were excluded from the study due to their potentially high prior knowledge about the research material used, in order to control for possible influences of prior knowledge. Participants wearing dark makeup in the eye area were also excluded from the study because in this case it would have been impossible to measure accurate eye-tracking data. Also, persons who participated in earlier experiments with the same material were excluded. Participants wearing glasses had to take them off and eyeglass lenses in approximately the same diopter strength as the lenses of their original glasses (divergence of a maximum of 0.25 diopters) were chosen to insert into the eye-tracking glasses. The participants were from a wide range of disciplines (e.g., medicine, law, psychology, physics, economics). From the *n* = 70 recruited participants, three had to be excluded from the analysis because they were familiar with one of the two test paintings. Six participants could not be eye-tracked with enough accuracy (eye-tracking ratio < 80%) or showed other technical problems with the eye-tracker and were therefore excluded from the analysis. Three participants were excluded because they did not speak German on a native speaker level, and finally, three participants were excluded because they did not follow the instructions to concentrate and do their tasks. Therefore, *n* = 55 participants remained for the analyses, including 39 female (70.9%) and 16 male (29.1%) participants. They were aged between 19 and 35 years (*M* = 23.96, *SD* = 3.78). Participants were randomly assigned to the two conditions LDI integrated (*n* = 26) and LDI separated (*n* = 29).

### Design

For all dependent variables, one-factorial ANCOVAs with the between-subjects factor audio explanation format (LDI integrated vs. LDI separated) were calculated.

### Material

Participants wore Tobii Pro Glasses 2, a 50 Hz mobile eye-tracking system, measuring their gazes on the presented learning material. Recorded gazes were analysed with the software Tobii Pro Lab (Analyzer Edition).

The learning material was presented on a 75-inch Sony Bravia FW-75 × 8570 C LED monitor (4 K, 3840 × 2160 px) with integrated speakers. The recognition test, the questions on demographics, exclusion criteria, and the control variables interest in history and interest in art were presented with the software PsychoPy version 3.2.4 on a laptop with integrated keyboard, mouse, and external speakers.

Four history paintings were presented on the computer screen together with a corresponding audio text explanation (all included within an mp4 video file). Two of them were used as flanking paintings (“The Death of Socrates” by Jacques Louis David and “The Proclamation of the German Emperor” by Anton von Werner) and two as testing material (“Washington Crossing the Delaware” by Emanuel Leutze and “The Death of General Wolfe” by Benjamin West). The order of the two test paintings alternated between participants in a counterbalanced way.

The audio explanations were spoken by a female speaker and initially informed the participants about the painting’s title, materiality of the painting, name and nationality of the painter, year of origin, and topic of the painting. Then, eight picture elements were explained by describing three of their details visible in the painting and interpreting each of these three details. In the LDI integrated condition the description of each detail was immediately followed by its interpretation, both either connected, for example, by argument overlaps in the form of one and the same noun, known synonyms, meta category words, or by semantically related phrases between the descriptive and the interpretation sentence. In the LDI separated condition in which descriptions and interpretations were separated, the details were mentioned one after another, followed by their interpretations as a separate subsequent block. Hereby, description and interpretation sentences were also connected either by argument overlaps in the form of one and the same noun, known synonyms, meta category words, or by semantically related phrases between the descriptive and the interpretation sentences, but across several - and not between - successive sentences. The audio explanations concluded with a short description of how the depicted historical events ended. The length of the audio explanations was equal for the LDI integrated and LDI separated versions: 08:04 min for the Leutze painting and 07:36 min for the West painting.

### Measures

**Interest in History and Interest in Art.** Interest in history and interest in art were measured as control variables each with one item (“How much are you interested in history?”, “How much are you interested in art?”) and a 6-point Likert scale ranging from (1) “not at all” to (6) “very much”.

**Fig. 1:**
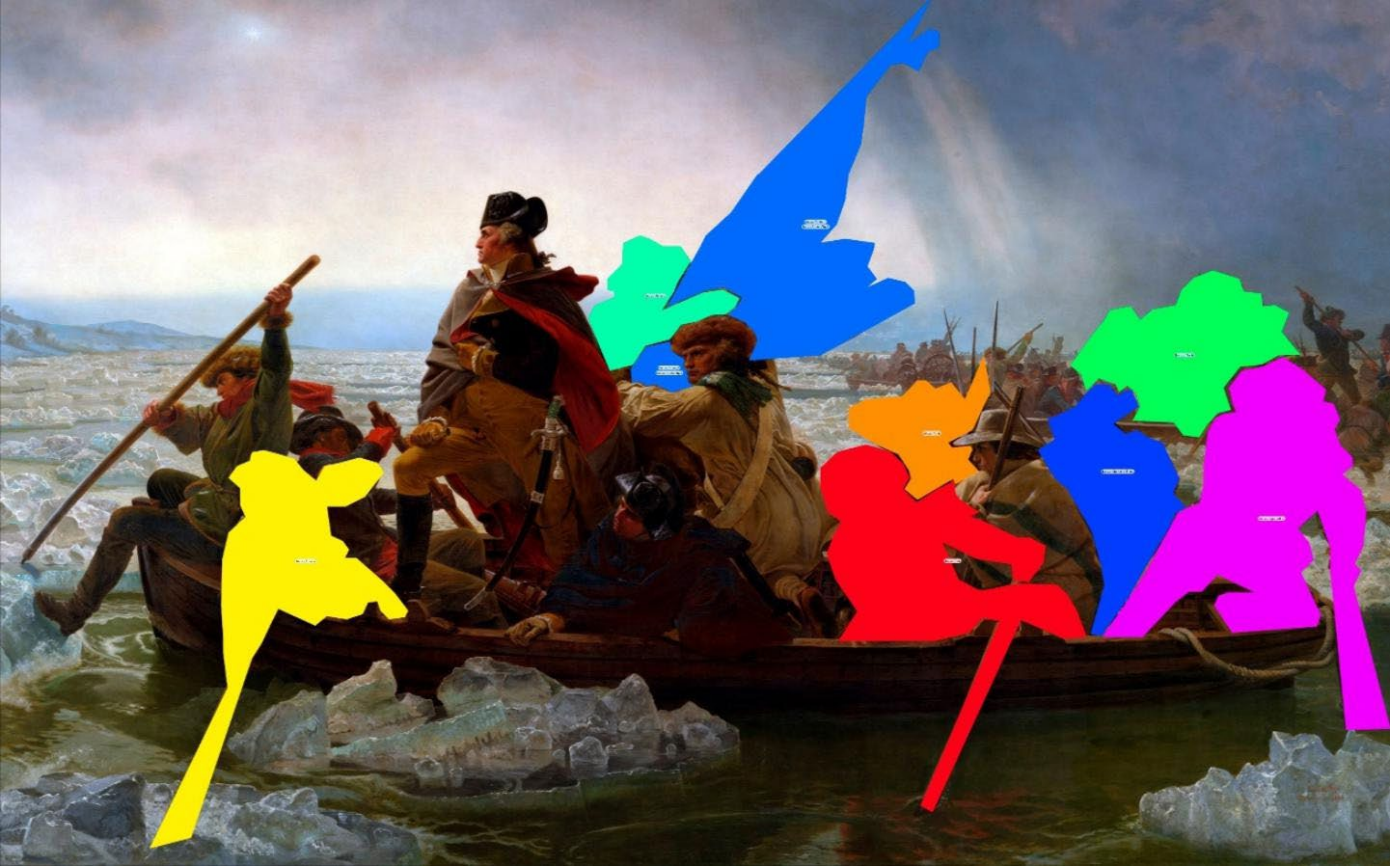
Areas of interest (AOIs) in the Leutze painting “Washington Crossing the Delaware”. *Note*. The picture of the painting “Washington Crossing the Delaware” by Emanuel Leutze was derived from https://en.wikipedia.org/wiki/George_Washington%27s_crossing_of_the_Delaware_River#/media/File:Washington_Crossing_the_Delaware_by_Emanuel_Leutze,_MMA-NYC,_1851.jpg on 31.01.2022. Licensing: public domain

**Fixation Times.** Fixation times [in seconds] on the areas of interest (AOI) of the referred-to picture elements during the time of their interpretations plus 500ms in the audio explanation were calculated. Sum scores were calculated across all picture elements and test paintings. As an example, the AOIs in the Leutze painting “Washington Crossing the Delaware” are presented in Fig. 1.

**Cued Recall Test.** Memory for details and interpretations of the picture elements as well as their linkages was measured by a cued recall test. The participants were asked to write down and additionally visually sketch all information they remembered from the paintings and the audio explanations with regard to particular picture elements that were cued by naming them in written form. Descriptions and drawings were considered complementary for the ratings. Participants received one point if they recalled a detail, an interpretation, or a linkage correctly. Per picture element, a maximum of three details, three interpretations, and three linkages was possible. Ratings were done by two independent raters. Interrater reliability (Krippendorff’s alpha) was α = 0.90. In cases in which these raters did not agree, a third rater made the decision. Separate sum scores were calculated for recall of details, recall of interpretations, and recall of linkages across all eight picture elements and test paintings, each ranging between 0 and 48 points.

**Recognition Task.** Memory of linkages between the described details of the picture elements and their corresponding interpretations was additionally measured by a recognition task in which a picture element was presented visually to the participants together with an audio statement about its interpretation. For each of the two test paintings, 24 pairs of picture detail and interpretation matched, while 16 pairs of picture element and interpretation did not. In half of the mismatch trials, interpretations and picture details were from the same test painting and in the other half of the mismatch trials, interpretations and picture details were from different test paintings. The sequence of all pairs was randomised across pictures. The participants had to decide via keypress whether the picture elements and the audio statements matched or not. They received one point for every correctly identified match (hit). The proportions of hits were calculated across all trails of matches and test paintings, ranging from 0 to 1 point.

### Procedure

The participants took part in the experiment in single sessions. They were welcomed, signed the consent form, and were then equipped with mobile eye-tracking glasses which first had to be calibrated. The participants were instructed that they would be presented with a video about four paintings, that the video would last approximately 30 min, that they would be asked questions about the video afterwards, and that while watching the video they should stay within the marked area of 1.70 × 1.70 m in front of the monitor. They were instructed that they were free to move within this area and that they were also allowed to sit on the barstool placed within the marked area. The participants then viewed the four paintings with accompanying audio explanations while their gazes were tracked by the eye-tracking system. Afterwards, they took off the eye-tracking glasses and did a filler task in the form of a memory puzzle for eight minutes in order to inhibit further memorization of the previous paintings and audio explanations. Then, the participants did the cued recall tasks about the Leutze and the West painting in the same order as the presentation of the paintings, each limited to ten minutes. Afterwards, they were seated in front of a laptop to do the recognition task and provide demographics (age, gender, field of study/profession), give answers to exclusion criteria and to their interest in history and art. Finally, the participants were thanked and paid for their participation. Each study sessions lasted approximately 75 minutes. The study received institutional research ethics committee approval.

### Data Analyses

Analyses were calculated with SPSS version 25. Post-hoc tests of all the calculated ANCOVAs were Bonferroni corrected to control for type 1 errors. Exploratory mediation analyses were calculated using PROCESS version 3.5.3 (Hayes, 2018). Effect sizes are interpreted according to Cohen (1988) who interprets *d* < 0.500 as a small effect, 0.500 < *d* < 0.800 as an intermediate effect, and *d* > 0.800 as a large effect, and eta squared values of η^2^ < 0.060 as small effect, 0.060 < η^2^ < 0.14 as intermediate effect, and η^2^ > 0.14 as large effect.

## Results

Interest in art was equally distributed between the LDI integrated (*M* = 2.81, *SD* = 1.20) and the LDI separated (*M* = 3.21, *SD* = 1.15) condition, *t*(53) = 1.26, *p* = .213, *d* = 0.34, 95% CI [-0.236, 1.034]. However, the participants in the two conditions significantly differed with regard to their interest in history, *t*(53) = 2.27, *p* = .028, *d* = 0.60, 95% CI [0.070, 1.148]. Those participants in the LDI separated condition (*M* = 3.72, *SD* = 0.92) had a significantly higher interest in history than the participants in the LDI integrated condition (*M* = 3.12, *SD* = 1.07). Therefore, interest in history was included as covariate into all our analyses.

**Fig. 2:**
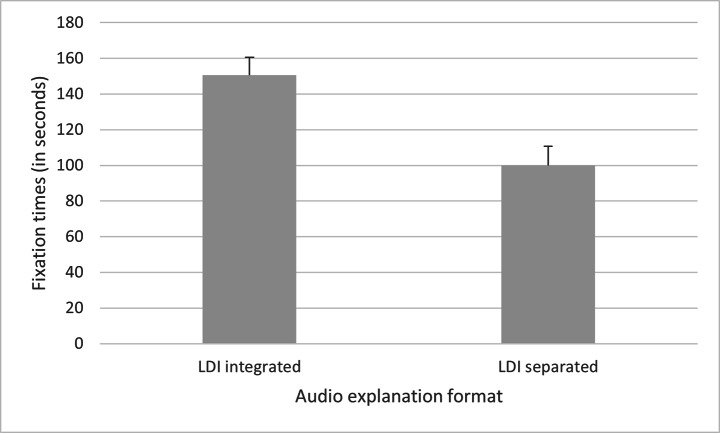
Differences in fixations times (in seconds) between the LDI integrated and the LDI separated audio explanation format. *Note*. Standard errors are in both directions, although only the positive ones are depicted.

A one-factorial analysis of variance (ANCOVA) with the between-subjects factor audio explanation format (LDI integrated vs. LDI separated) and the covariate interest in history was calculated with regard to fixation times on referred-to AOIs during interpretation sentences plus 500ms. The results showed a significant main effect of audio explanation format, *F*(1,52) = 10.75, *p* = .002, η_p_^2^ = 0.171, 95% CI [19.82, 82.33]. During interpretations in the audio explanations, AOIs of referred-to picture elements were significantly longer fixated in the LDI integrated condition (*M* = 150.55, *SD* = 51.14) than in the LDI separated condition (*M* = 99.92, *SD* = 57.42), confirming hypothesis H1. This is shown in Fig. 2. Descriptive statistics in the two conditions are presented in Table 1, as are the descriptive statistics of all other dependent variables in the two conditions of the following analyses.

**Table 1:** *Mean values and standard deviations of the dependent variables in the two conditions of each analysis*

Analysis	Integrated Condition		Separated Condition	
	*M*	*SD*	*M*	*SD*
*t*-Test: interest in art (1-item-value ranging from 1–6 points)	2.81	1.20	3.21	1.15
*t*-Test: interest in history (1-item-value ranging from 1–6 points)	3.12	1.07	3.72	0.92
One-factorial ANCOVA: fixation times (in seconds)	150.55	51.14	99.92	57.42
One-factorial ANCOVA: time to first fixation (in seconds)	1.28	0.42	2.58	0.86
One-factorial ANCOVA: cued recall of descriptions (sum scores ranging from 0 to 48 points)	19.19	5.38	17.93	6.35
One-factorial ANCOVA: cued recall of interpretations (sum scores ranging from 0 to 48 points)	13.00	5.25	13.34	6.69
One-factorial ANCOVA: cued recall of linkages (sum scores ranging from 0 to 48 points)	6.42	3.57	5.38	3.09
One-factorial ANCOVA: proportion of hits in recognition test (scores ranging from 0 to 1 point)	0.84	0.11	0.78	0.13

**Fig. 3:**
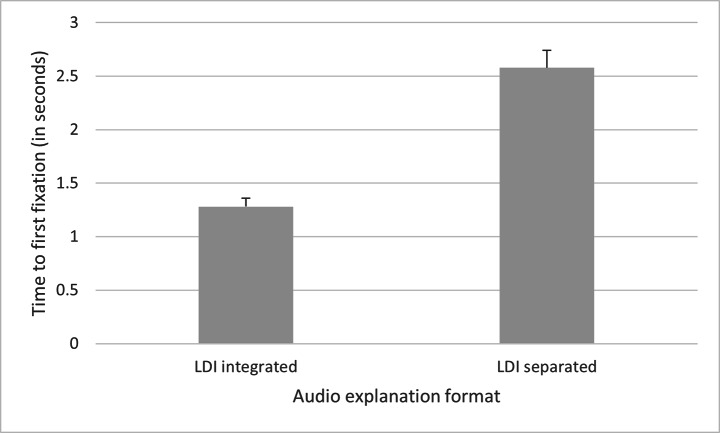
Differences in time to first fixation (in seconds) between the LDI integrated and the LDI separated audio explanation format. *Note*. Standard errors are in both directions, although only the positive ones are depicted

Exploratorily, we also calculated a one-factorial analysis of variance (ANCOVA) with the between-subjects factor audio explanation format (LDI integrated vs. LDI separated) and the covariate interest in history with regard to the time [in seconds] to first fixation of the corresponding picture element during the interpretation phases. The results showed a significant effect for audio explanation format, *F*(1, 52) = 41.56, *p* < .001, η_p_^2^ = 0.444, 95% CI [0.87, 1.65]. Time to first fixation was significantly shorter in the LDI integrated condition (*M* = 1.28, *SD* = 0.42) than in the LDI separated condition (*M* = 2.58, *SD* = 0.86). This is shown in Fig. 3. However, it has to be noted, that in this analysis, variances were heterogenous between the conditions and therefore these results have to be interpreted with caution.

**Fig. 4:**
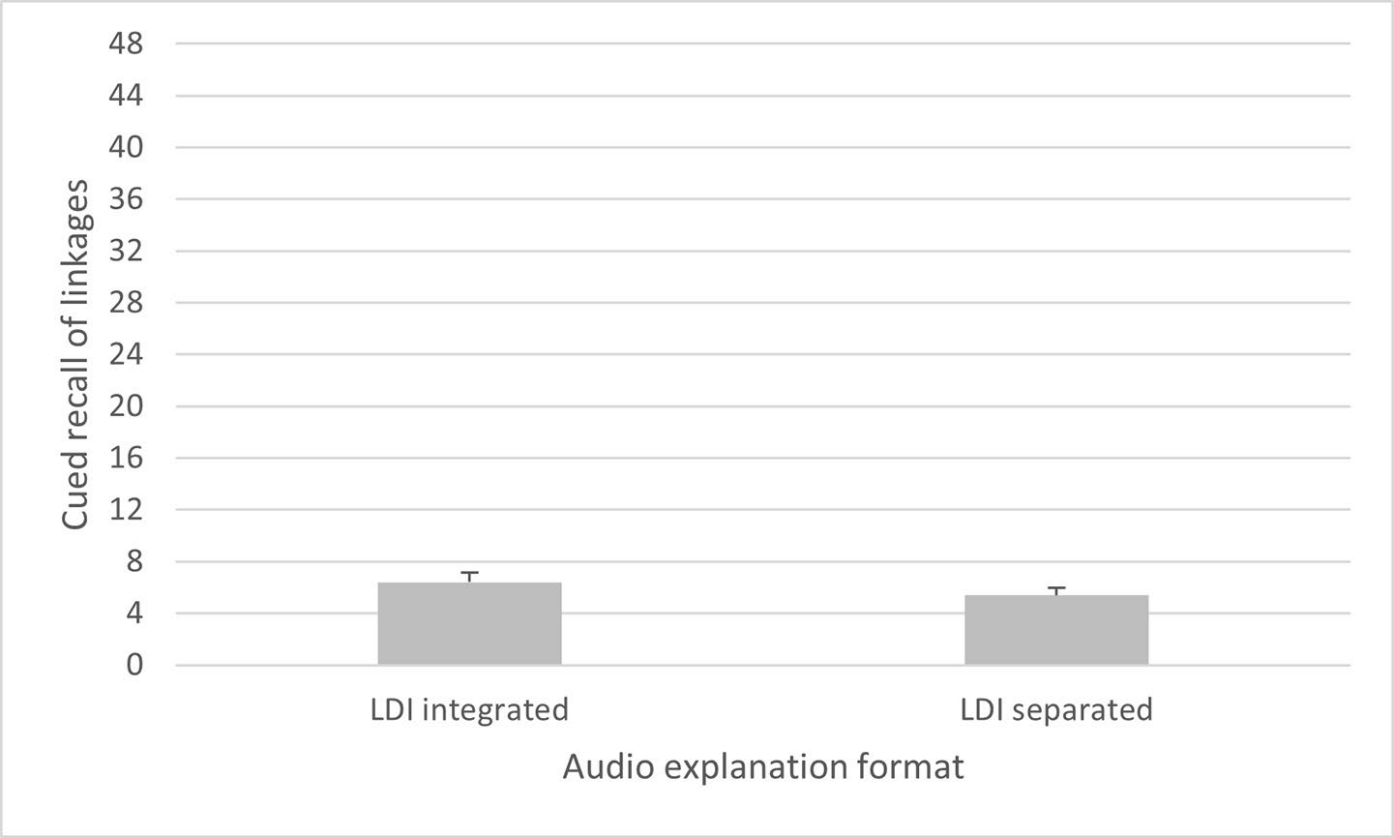
Differences in cued recall of linkages between the LDI integrated and the LDI separated audio explanation format. *Note*. Standard errors are in both directions, although only the positive ones are depicted

One-factorial analyses of variance (ANCOVA) with the between-subjects factor audio explanation format (LDI integrated vs. LDI separated) and the covariate interest in history were calculated with regard to the cued recall of descriptions of details of picture elements, cued recall of interpretations, and cued recall of linkages between descriptions of details of picture elements and their interpretations. The results regarding the cued recall of descriptions of details of picture elements showed no main effect of audio explanation format, *F*(1,52) = 3.07, *p* = .086, η_p_^2^ = 0.056, 95% CI [-0.393, 5.814], with no significant difference between the LDI integrated condition (*M* = 19.19, *SD* = 5.38) and the LDI separated condition (*M* = 17.93, *SD* = 6.35). With regard to cued recall of interpretations, there was also no main effect for audio explanation format, *F*(1,52) = 0.21, *p* = .649, η_p_^2^ = 0.004, 95% CI [-2.558, 4.068], with no significant difference between the LDI integrated condition (*M* = 13.00, *SD* = 5.25) and the LDI separated condition (*M* = 13.34, *SD* = 6.69). This does not support hypothesis H2. With regard to cued recall of linkages between descriptions of details of referred-to picture elements and their interpretations, however, results showed a significant main effect of audio explanation format, *F*(1,52) = 4.07, *p* = .049, η_p_^2^ = 0.073, 95% CI [0.010, 3.557]. Linkages between descriptions and interpretation of referred-to picture elements were better recalled in the LDI integrated condition (*M* = 6.42, *SD* = 3.57) than in the LDI separated condition (*M* = 5.38, *SD* = 3.09), confirming hypothesis H3. This is shown in Fig. 4.

**Fig. 5:**
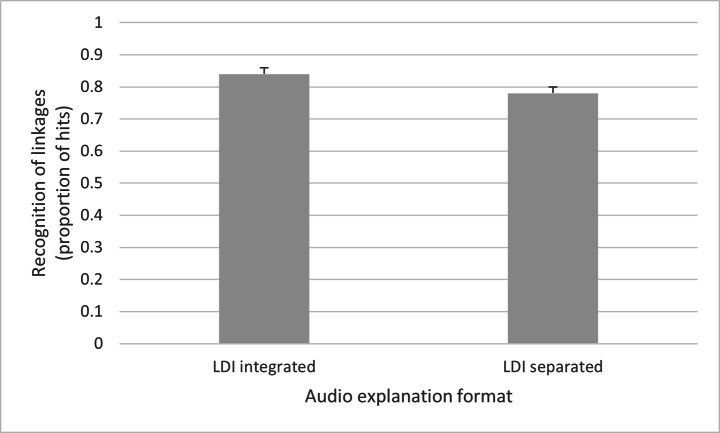
Differences in recognition of linkages (proportion of hits) between the LDI integrated and the LDI separated audio explanation format. *Note*. Standard errors are in both directions, although only the positive ones are depicted

A one-factorial analysis of variance (ANCOVA) with the between-subjects factor audio explanation format (LDI integrated vs. LDI separated) and the covariate interest in history was calculated with regard to the proportion of hits in the recognition test. The results showed a significant main effect of audio explanation format, *F*(1,52) = 5.62, *p* = .022, η_p_^2^ = 0.098, 95% CI [0.012, 0.148]. There were significantly more correct identifications of linkages between picture elements and audio interpretation statements (hits) in the LDI integrated condition (*M* = 0.84, *SD* = 0.11) than in the LDI separated condition (*M* = 0.78, *SD* = 0.13). This is shown in Fig. 5.

**Fig. 6:**
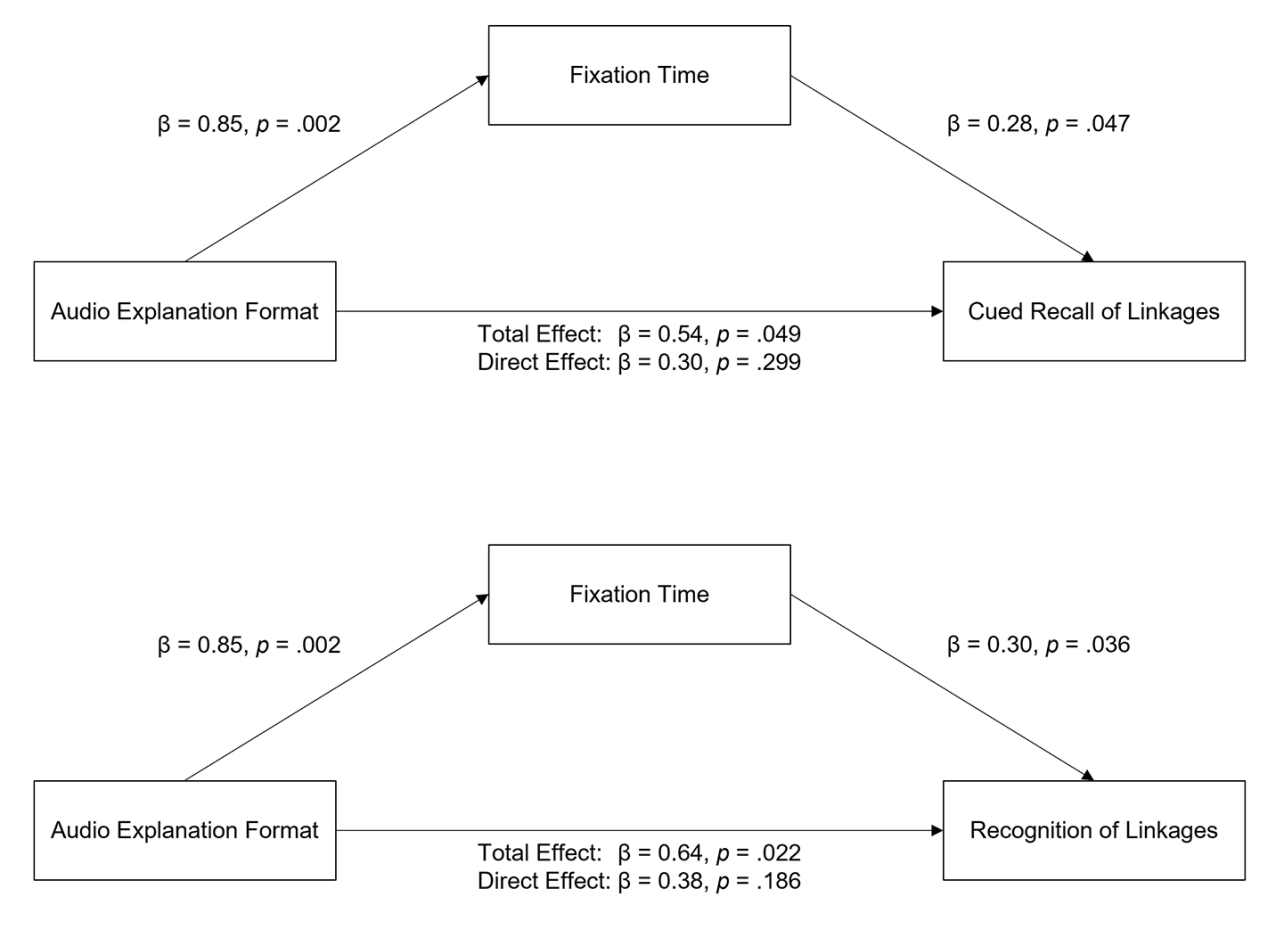
Standardized coefficients and p-values for the effects of audio explanation format (LDI integrated vs. LDI separated) on cued recall of linkages between descriptions of details of picture elements and their interpretations (above) and on the proportion of hits in the recognition test (below), both fully mediated by fixation time on the referred-to picture elements during interpretations of details of picture elements in the audio explanation

Exploratively, two mediation analyses were calculated with audio explanation format (LDI integrated vs. LDI separated) as the independent variable, fixation times on referred-to AOIs during interpretation sentences plus 500ms as mediator, interest in history as covariate, and either cued recall of linkages between descriptions of details of picture elements and their interpretations or proportion of hits in the recognition test as dependent variables. With regard to cued recall of linkages between descriptions of details of picture elements and their interpretations, the results show a significant model summary, *F*(3, 51) = 4.73, *p* = .006, *R²* = 0.22. Audio explanation format significantly predicted cued recall of linkages (*b* = 1.7839, *t* = 2.02, *p* = .049, 95% CI [0.0104, 3.5574], partially standardized total effect *c*_*ps*_ = 0.5351) as well as fixation times (*b* = 51.0770, *t* = 3.28, *p* = .002, 95% CI [19.8205, 82.3335]). When audio explanation format and the mediator fixation time were considered simultaneously, the direct effect of audio explanation format on cued recall of linkages was not significant (*b* = 0.9900, *t* = 1.05, *p* = .299, 95% CI [-0.9029, 2.8828], partially standardized direct effect *c’*_*ps*_ = 0.2970), whereas the effect of the mediator was significant (*b* = 0.0155, *t* = 2.03, *p* = .047, 95% CI [0.0002, 0.0309]). Using 5000 bootstrap resamples, the indirect effect of audio explanation format on cued recall of linkages by the mediator fixation times was significantly different from zero, partially standardized indirect effect *ab*_*ps*_ = 0.2382. With regard to the proportion of hits in the recognition test, results also show a significant model summary, *F*(3, 51) = 4.34, *p* = .009, *R²* = 0.20. Audio explanation format significantly predicted the proportion of hits in the recognition test (*b* = 0.0800, *t* = 2.37, *p* = .022, 95% CI [0.0123, 0.1477], partially standardized total effect *c*_*ps*_ = 0.6368) as well as fixation times (*b* = 51.0770, *t* = 3.28, *p* = .002, 95% CI [19.8205, 82.3335]). When audio explanation format and the mediator fixation time were considered simultaneously, the direct effect of audio explanation format on the proportion of hits in the recognition test was not significant (*b* = 0.0481, *t* = 1.34, *p* = .186, 95% CI [-0.0239, 0.1201], partially standardized direct effect *c’*_*ps*_ = 0.3829), whereas the effect of the mediator was significant (*b* = 0.0006, *t* = 2.15, *p* = .036, 95% CI [0.0000, 0.0012]). Using 5000 bootstrap resamples, the indirect effect of audio explanation format on the proportion of hits in the recognition test by the mediator fixation times was significantly different from zero, partially standardized indirect effect *ab*_*ps*_ = 0.254. The effects of audio explanation format on cued recall of linkages and the proportion of hits in the recognition test were therefore both fully mediated by fixation times (see Fig. 6).

## Discussion

The present study examined the influence of audio explanation structure on attention and memory for pictorial content accompanied by spoken explanations. Such material is often used in digital scenarios with complex pictures, such as online courses, educational software, virtual exhibitions, or digital collections. Audio explanation structure was manipulated by different arrangements of localization - description - interpretation (LDI structures). Audio explanations in which the localization and description of details of each picture element were presented together with its interpretation (LDI integrated structure) established local coherence between these text parts. They were compared to audio explanations in which localization and description of the details of the picture elements were separated from their interpretation, therefore requiring listeners to establish global coherence between both text parts (LDI separate structure). Differences between these conditions were measured with regard to fixation times on the referred-to picture elements during the time of their interpretations in the audio explanation and with regard to memory for details and interpretations of the picture elements as well as their linkages. Interest in art was equally distributed between the conditions and since interest in history was not, interest in history was considered as covariate in the analyses.

The results showed that the referred-to picture elements were fixated significantly faster and longer during the interpretation parts of the audio explanation if localization, description, and interpretation were integrated (LDI integrated structure) compared to audio explanations in which localization, description, and interpretation were separated (LDI separated structure). This was a large effect, which is in support of hypothesis H1. If the interpretation of a picture element immediately followed its localization and description, the viewers took less time to fixate the element at the beginning of the interpretation part. This was also a large effect, although it has to be interpreted with caution due to the statistical reasons mentioned above. If it can be interpreted, this early fixation indicates that the process of searching the picture element referred to verbally was more efficient in the LDI integrated condition. This more efficient search may have saved time, which was then available for better learning, as indicated by longer fixation times than in the LDI separated condition.

In addition, while the LDI integrated condition and the LDI separated condition did not show significant differences in recall of descriptions and interpretations themselves, linkages between descriptions and interpretation of the picture elements referred to were both recognized and recalled better in the LDI integrated condition. These were medium effects and hence, hypothesis H3 on the memory of linkages between descriptions and interpretations was confirmed by the findings, but not hypothesis H2 on the memory of interpretations themselves. The results indicate that developing a mental representation which relates interpretations to details of picture elements was easier when the descriptions and interpretations were presented in close succession than in separate blocks of the audio explanation. Local audio explanation coherence with LDI integrated structure compared to global audio explanation coherence with LDI separate structure may have enhanced fluent processing and comprehension not only by prohibiting effortful visual search processes to allocate visual attention onto the referred-to picture elements in the interpretation phase (as shown by the present results with regard to fixation times), but also by prohibiting more effortful global inferences in favour of less effortful local inferences. This may have led to a better linkage of description and interpretation of details of picture elements and therefore to a better attribution of meaning to picture elements and picture comprehension. The results are thus in line with previous text comprehension research showing that establishing global coherence is more effortful than establishing local coherence (Albrecht & Myers, 1995; Long & Chong, 2001; McKoon & Ratcliff, 1992) and with most of the prior text comprehension research showing effects of local text coherence on comprehension (Degand & Sanders, 2002; Sanders et al., 2007; van Silfhout et al., 2015; Wittwer & Ihme, 2014). The finding that only the memory of linkages between descriptions and interpretations - but not the memory of the interpretations themselves - was affected by LDI structure indicates that interpretations may have been learned and remembered independent of their local or global connection with the picture details.

Interestingly, the effects of LDI structure on the memory of linkages were fully mediated by visual attention, indicated by fixation times on the referred-to picture elements during the interpretation phases in the audio explanation. In particular, with regard to the cued recall task, this indicates that, in multimedia picture comprehension scenarios, the correct attribution of meaning to picture elements cannot be provided solely by locally coherent audio explanations that closely link descriptions and interpretations of details of picture elements but requires visual attention on the respective picture elements. The findings indicate that an integrated LDI structure offers advantages in terms of dual encoding because it keeps the attentional focus on the relevant picture element during the description as well as the interpretation part of the verbal explanation. Thus, picture elements can be linked to both description and interpretation to form a mental representation. In contrast, when localization and description are separated from interpretation, the attentional focus on the picture element established during localization and description is dissolved and recipients must instead either reorient their visual attentional focus when the interpretation part begins, which may fail, or they have to retrieved pictorial information from long-term memory, if available, in order to integrate it with the description and interpretation in working memory. Retrieval of picture details from long-term memory as well as relocation of visual attention are both resource consuming processes that reduce cognitive capacity available to integrate description, interpretation, and pictorial information and store it in long-term memory as described by the cognitive theory of multimedia learning (Mayer, 2014). In other words, due to the longer and faster visual attention to the picture details during the interpretation phase in the integrated LDI condition compared to the separate LDI condition, the integrated LDI condition benefits more from dual coding than the separate LDI condition. By means of these dual coding processes (Glaser et al., 2020a; Glaser & Schwan, 2015; Mayer, 2014) interpretations are linked not only to the auditive description but also to audio-visual picture details. This is in line with previous research by Tibus et al., (2013) who showed that pictorial representations can aid learners in building inferences and therefore comprehension. It highlights furthermore the importance of the attention guiding role of audio explanations in multimedia learning scenarios already postulated by Glaser and Schwan (2015), as well as the importance of localization information in audio explanations that helps the learners to guide their attention across pictures. Future research should therefore put more emphasis on examining the interplay between text coherence and dual coding processes in multimedia learning scenarios with a particular focus on the role of visual attention.

There are also certain limitations of the present study. First, due to COVID-19 pandemic related restrictions, with 55 participants the sample size was rather small. Nevertheless, the study was able to uncover substantial effects in accordance with the prespecified hypotheses. Further, the present study was carried out in a laboratory with a rather homogenous group of participants. Future studies should replicate the effects found in the present study also in the field, for example, when learning with educational videos on YouTube or with pictures of paintings and accompanying audio explanations on museum websites. In field studies, also a broader variety of recipients with different learner characteristics would be observable and effects may be differentiated more strongly between different types of learners. For example, instead of excluding participants with high prior knowledge (participants studying art, art-history, or history or knowing at least one of the analysed paintings) as was done in the present study, prior knowledge could be treated as a potential influencing factor and be measured explicitly like in the study by McNamara et al. (1996).

The present study examined, for the first time, text coherence effects with audio explanations in a complex multimedia learning scenario with a focus on picture comprehension. The pattern of results provides some important practical implications on how educational videos, and audio text and picture combinations on websites, and also audio guides in museums, should be designed in order to facilitate picture comprehension by guiding the viewers’ attention through the picture, helping them not only to notice important elements and direct visual attention to them, but also to attribute meaning provided via audio interpretations to picture elements (Schwan et al., 2018; Webb & Mann, 2014). More specifically, audio explanations with LDI integrated structure as often used for audio guides (Popp, 2013) seem to be more appropriate than LDI separated structures that have often been established in the field of arts and culture (Bauer & Schwan, 2018; Panofsky, 1975) as well as in school settings (Tishman, 2017). While to the best of our knowledge structural analyses of expository texts accompanying pictorial material (like maps or infographics) are lacking, we speculate that the present findings regarding processing differences between LDI integrated and LDI separated structure might also be relevant for scientific content, which should be addressed in future studies. In addition, by generalizing previous findings from text comprehension research most often examining written texts to a multimedia learning situation with audio explanations and by distinguishing audio text coherence and dual coding mechanisms as explanations for the effects of audio explanation design on picture comprehension, the present study also contributes to a better theoretical understanding of meaning making with digital pictorial material.
